# Atypical initial presentation of Takayasu arteritis as isolated supra-valvular aortic stenosis

**DOI:** 10.1186/s13019-016-0408-0

**Published:** 2016-01-19

**Authors:** Do Yeon Kim, Hwan Wook Kim

**Affiliations:** Department of Thoracic and Cardiovascular Surgery, Seoul St. Mary’s Hospital, College of Medicine, The Catholic University of Korea, 222 Banpo-daero, Seocho-gu, Seoul 137-701 Republic of Korea

**Keywords:** Takayasu arteritis, Supra-valvular aortic stenosis, Bentall opearation

## Abstract

**Background:**

Among the vascular involvements of Takayasu arteritis, a supra-valvular aortic stenosis has been reported very rarely. We report a case of surgically corrected, supra-valvular aortic stenosis caused by Takayasu arteritis.

**Case presentation:**

A 32-year-old female was diagnosed with supra-valvular aortic stenosis by transthoracic echocardiography for the evaluation of cardiac murmur with constitutional symptoms. Under the impression of non-familial sporadic type of supra-valvular aortic stenosis, surgical correction was performed. However, after 1 year from the operation, we could know the cause of her disease through the findings of computed tomographic aortography that Takayasu arteritis was suspected.

**Conclusions:**

Takayasu arteritis should be considered in adult female patients presenting supra-valvular aortic stenosis with constitutional symptoms, even if no typical features of vascular involvement.

**Electronic supplementary material:**

The online version of this article (doi:10.1186/s13019-016-0408-0) contains supplementary material, which is available to authorized users.

## Background

Takayasu arteritis (TA) is a chronic inflammatory vascular disease affecting the aorta, its major branches and pulmonary arteries. TA typically leads to progressive fibrosis in arterial wall resulting in stenosis or occasionally triggers destruction in the arterial media layer that drives aneurysmal change [1]. Isolated focal supra-valvular ascending aortic stenosis is a very rare vascular involvement of TA [2]. Therefore, it can be confused with the non-familial, sporadic type of supra-valvular aortic stenosis.

We report an adult female patient treated initially by surgery (Bentall procedure with aortic root augmentation) under the impression of non-familial, sporadic type of supra-valvular aortic stenosis, who was subsequently diagnosed as TA by a meticulous review of medical records and new vascular presentations.

## Case presentation

**Fig. 1:**
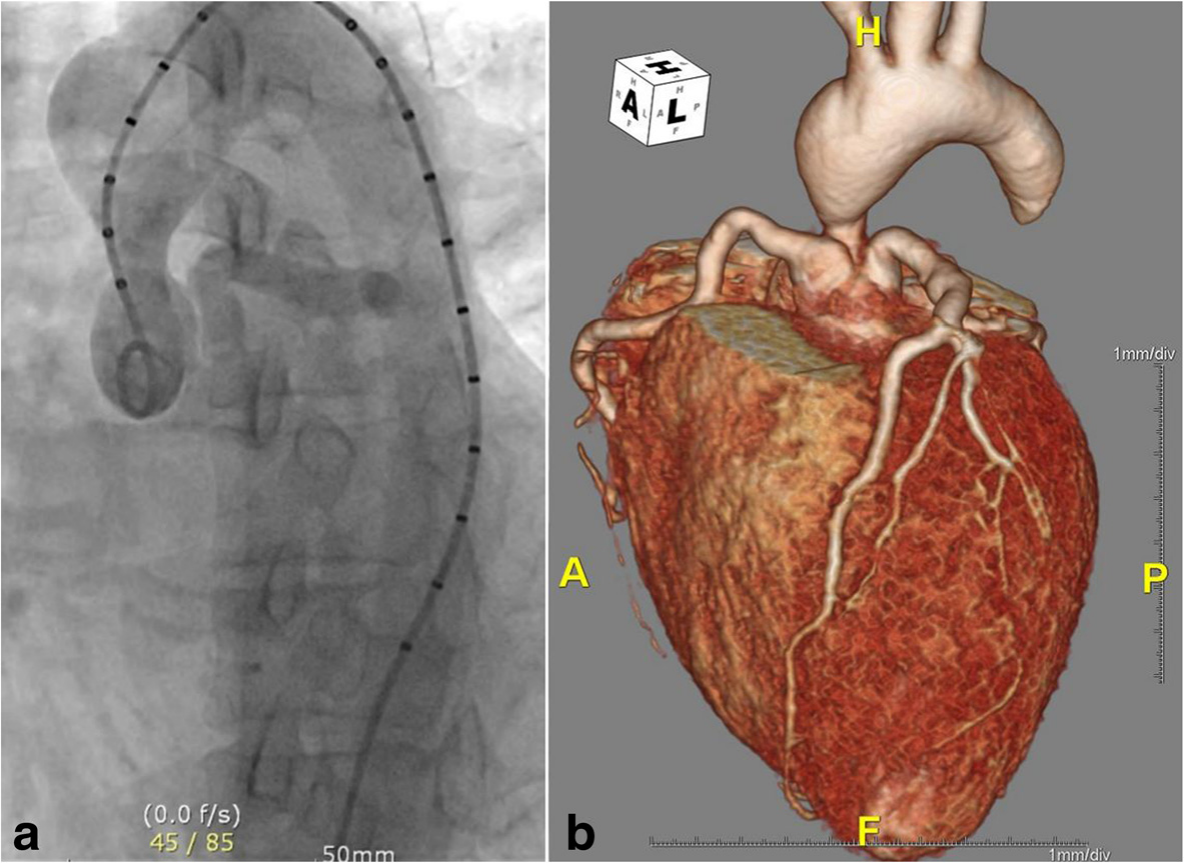
**a** Aortography showing a focal, hourglass-type supra-valvular aortic stenosis with normal morphology of aortic arch and descending thoracic aorta. **b** Three-dimensional reconstruction imaging of computed tomography showing the spatial and anatomical relationship of the stenotic aortic root and coronary orifices

A 32-year-old Korean female was referred for progressive symptoms characterized by mild fever (37.8 °C) and dyspnea on exertion. One-month before, she had experienced the first instance of numbness in the left fingers and had recovered without any treatment. The patient was an elementary school teacher with normal appearance. She had given birth to a baby without any difficulties 2 years previously. On admission, a parasternal systolic murmur was revealed by physical examination. Blood test showed mild leukocytosis (14.6 X 10^9^ cells/L), elevated erythrocyte sedimentation rate (52 mm/h), and C-reactive protein (4.64 mg/dl). Transthoracic echocardiography revealed a mean and peak pressure gradient across the aortic root of 75 mmHg and 130 mmHg, respectively, with resultant left ventricular hypertrophy (septal wall thickness = 17 mm; left ventricular posterior wall thickness = 15.5 mm). In addition, three thickened aortic valvular cups without limited movement resulting in mild aortic regurgitation were detected. Aortography showed a focal, hourglass-type supra-valvular aortic stenosis with normal morphology of aortic arch and descending thoracic aorta (Fig. 1a, Additional file 1). Electrocardiogram-gated computed tomographic aortography revealed the more accurate spatial and anatomical relationship of the stenotic ascending aorta and aortic root including the orifice of coronary arteries (Fig. 1b). Moreover, left ventricular apical aneurysm was found with mural thrombus (Additional file 2) despite the absence of coronary abnormalities on angiography. Consecutive blood cultures were devoid of microorganisms. Under the impression of non-familial sporadic type of supra-valvular aortic stenosis, surgical correction was performed.

**Fig. 2:**
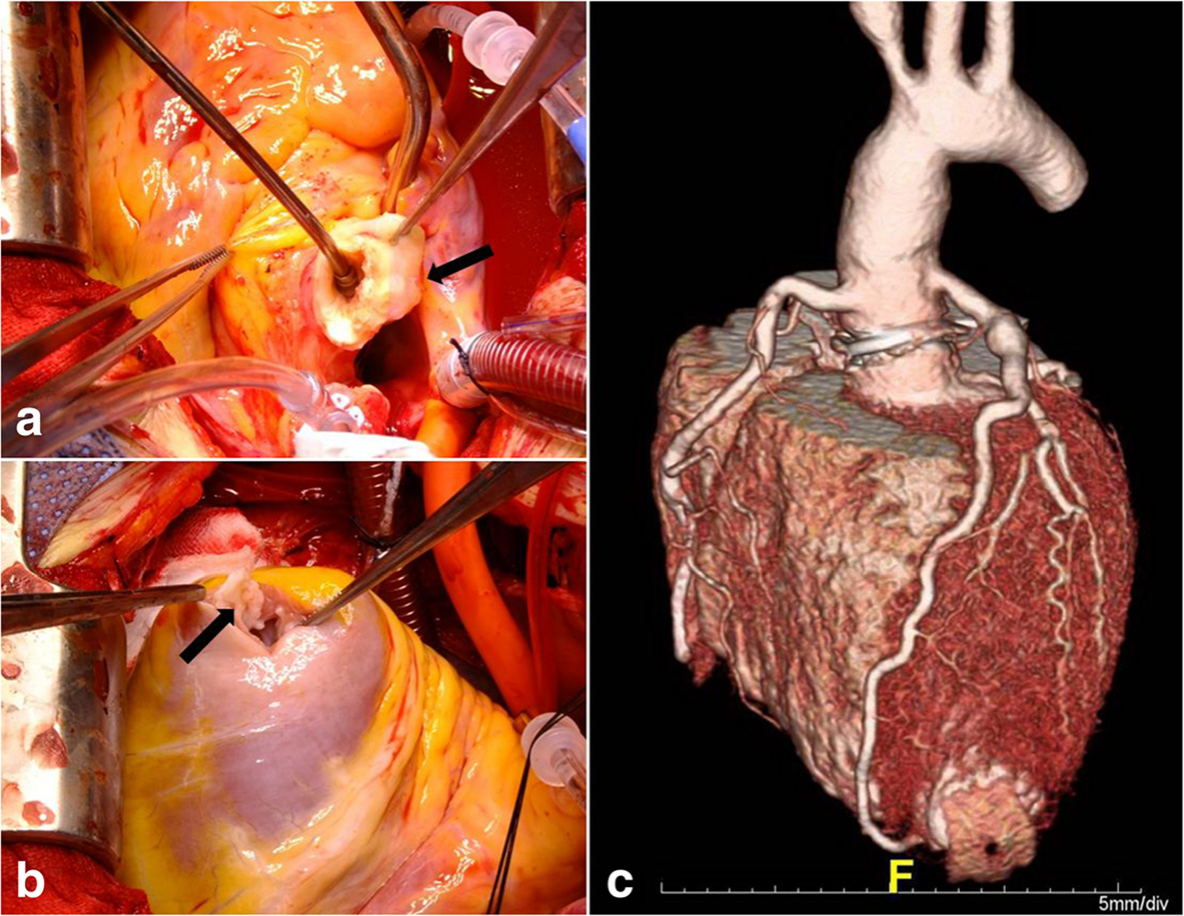
Intraoperative exploration showing a severe stenotic change with thickening of the ascending aortic wall (**a**, *arrow*) and the aneurysmal change (mural thrombus was not presented because of evacuation with suction device) of the left ventricular apex (**b**, *arrow*). Postoperative 1-year, a three-dimensional computed tomography reconstruction showed the successful surgical repair of stenotic ascending aorta using an aortic valve graft and newly perceived disease of the innominate artery (**c**)

Through a median sternotomy, cardiopulmonary bypass was established with the use of the right axillary artery for arterial return through a side 8-mm graft and right atrium for venous drainage. The patient was gradually cooled to 25 °C and the distal ascending aorta was cross-clamped. For optimal myocardial protection, cold blood cardioplegia was infused with a retrograde pattern initially and a selective pattern after resection of the diseased aortic segment. Longitudinal aortotomy revealed severe stenotic wrinkling change of ascending aorta just above the dilated orifice of both coronary arteries without discrete sinus of Valsalva (Fig. 2a). Three cusps of the aortic valve had suffered dismorphic thickening without motion limitation, together with circumferential small aortic annulus. Separate left ventricular apical incision revealed extremely thin walled transmural scar with an intraventricular clot (Fig. 2b).

**Fig. 3:**
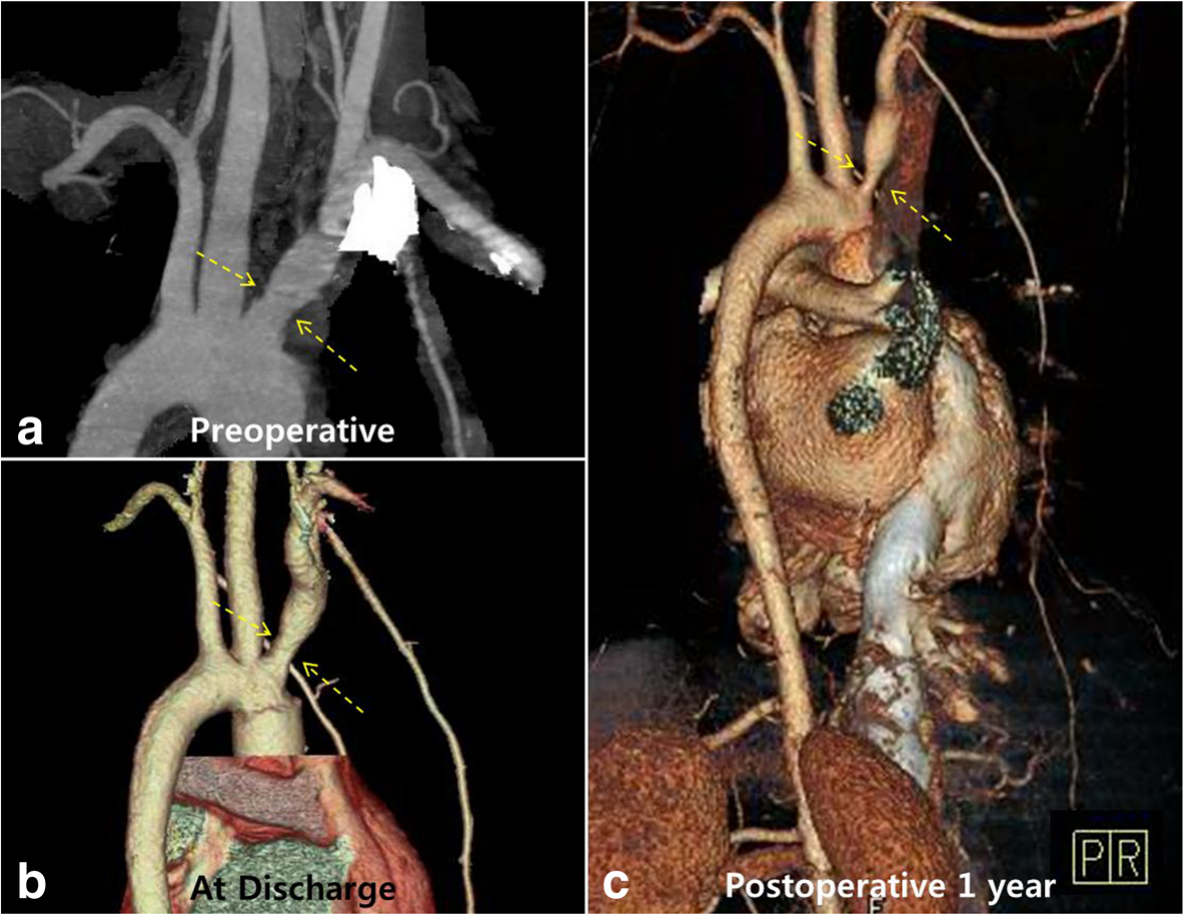
Serial review of perioperative imaging data revealed a subtle change of the innominate artery orifice (**a**, **b**, *arrow*) with small caliber of descending aorta (**c**)

Once evacuation of the mural thrombus in the left ventricular apex had been completed, the incision was closed with mattress sutures of 3–0 prolene buttressed by felt strips. Because of small aortic annulus with distorted cusps of aortic valve, aortic root replacement with an aortic valve graft (19-mm SJM regent valve; Saint Jude Medical, St. Paul, MN, USA) were performed after aortic root augmentation using Bovine pericardium according to the Manouguian method. Finally, the proximal aortic arch was anastomosed with the distal end of an aortic valved graft under bilateral selective cerebral perfusion through the right axillary artery and a separate perfusion catheter into the left common carotid artery. The patient was discharged without any complications 12 days postoperatively. Histopathologic examination revealed myxoid degeneration and fibrotic change of the diseased aortic wall. Genetic testing for supra-valvular aortic stenosis (fluorescent in situ hybridization test in the search for a microdeletion in the chromosome 7q11.23) was negative. A postoperative transthoracic echocardiogram showed normal mechanical aortic valve function without stenosis. Postoperative 1-year follow-up computed tomographic aortography revealed good contour of prosthetic materials located on the ascending aorta (Fig. 2c). However, a slightly aggravated stenotic lesion on innominate artery orifice and shrinkage of the entire abdominal aorta were found (Fig. 3).

## Discussion

TA, also known as pulseless disease, is first described by ophthalmologist Mikito Takayasu in 1908 [3]. This chronic, large vessel vasculitis predominantly affects females in their second and third decades of life, and usually involves large arteries like the aorta, supra-aortic branches, visceral branches, and pulmonary arteries. Constitutional symptoms that are also present include fever, weight loss, malaise, anorexia, and sweating [4]. Insidious progression of TA with diverse presentations frequently causes a delay in diagnosis of several months or years from the disease onset [5]. The lack of definitive objective assessments and no universally accepted pathophysiology of TA are other obstacles for optimal diagnosis. However, characteristic vascular abnormalities with resultant ischaemic symptoms for TA were well delineated in the diagnostic criteria defined by Ishikawa et al. in 1988 [6] and in classification criteria established by the American College of Rheumatology in 1990 [7]. The most common pattern of vascular abnormality is the stenotic involvement of the entire aorta from the aortic root to below the iliac bifurcations, with concomitant obstructive lesions of the first branched large arteries. The subclavian artery is the most commonly affected branched vessel, followed in order by the common carotid, renal, vertebral, and innominate arteries [2]. Isolated focal supra-valvular ascending aortic stenosis adjacent to the sinotubular junction without other vascular lesion, which resembles genetic supra-valvular aortic stenosis, is an extremely rare type of vascular involvement of TA.

Initially, the non-familial, sporadic type of supra-valvular aortic stenosis combined with infectious aortitis was suspected in this patient. Factors in this misdiagnosis included normal intelligence without facial abnormalities and stereotypical ‘hour-glass’ shaped ascending aortic stenosis. Even though no pathogen was detected in preoperative blood culture and postoperative tissue culture, constitutional symptoms of fever and myalgia disappeared after surgery. In addition, leukocytosis with elevated erythrocyte sedimentation ratio and C-reactive protein declined to normal range at discharge. However, the diagnosis was corrected at the 1-year follow-up outpatient visit.

Postoperative 1-year follow-up computed tomographic aortography revealed mild stenotic change in the innominate artery orifice and obvious shrinkage of the abdominal aorta. However, patient had remained symptom-free and the erythrocyte sedimentation ratio and level of C-reactive protein were normal with no leukocytosis. A re-examination of preoperative images revealed a subtle lesion of innominate artery orifice and relatively small, but still within the normal range, morphology of the abdominal aorta. At that time, TA was diagnosed according to the American College of Rheumatology criteria and the patient was referred to our rheumatology department for medical treatment focusing on immunity.

Apart from TA, the left ventricular apical aneurysm with mural thrombus was an interesting finding. This patient had a suspicious transient ischaemic symptom, or spontaneous remission of numbness in left fingers without affecting left subclavian artery. Subsequent imaging study of brain revealed no overt ischaemic lesion. Mural thrombus in the left ventricular apical aneurysm may have been responsible for ischemic presentation in the patient. We believe that long-standing intra-ventricular pressure overload was caused by supra-valvular aortic stenosis, which might have resulted in left ventricular apical ischaemia in a quiet way and an ultimate aneurysmal change [8].

## Conclusions

This case reminds us that TA should be considered in adult female patients presenting supra-valvular aortic stenosis with constitutional symptoms, even if no typical features of vascular involvement. Because of diverse manifestations and indolent encroachment of TA, high suspicion and vigilant surveillance including meticulous review of medical records are essential for correct diagnosis.

### Consent

Written informed consent was obtained from the patient for publication of this Case report and any accompanying images. A copy of the written consent is available for review by the Editor-in-Chief of this journal.

## Additional files

Additional file 1:
**Aortography showing a focal, hourglass-type supra-valvular aortic stenosis with normal morphology of the aortic arch and descending thoracic aorta.** (MP4 3404 kb) Additional file 2:
**Magnetic resonance imaging study showing mural thrombus in the left ventricular aneurysm. **(MP4 2342 kb)

## Abbreviation

TA: Takayasu arteritis
